# Effect of weight loss and liraglutide on neutrophil gelatinase-associated lipocalin levels among individuals with overweight and knee osteoarthritis: Exploratory analyses of a randomized controlled trial

**DOI:** 10.1016/j.ocarto.2024.100562

**Published:** 2025-01-03

**Authors:** Asbjørn Seenithamby Poulsen, Zara Rebecca Stisen, Marie Skougaard, Robin Christensen, Anders Overgaard, Henrik Gudbergsen, Stine Jacobsen, Andreas Peter Balslev-Clausen, Marius Henriksen, Lars Erik Kristensen, Henning Bliddal

**Affiliations:** a The Parker Institute, Copenhagen University Hospital – Bispebjerg and Frederiksberg, Denmark; b Department of Clinical Medicine, University of Copenhagen, Denmark; c Copenhagen Center of Translational Research, Copenhagen University Hospital – Bispebjerg and Frederiksberg, Copenhagen, Denmark; d Department of Clinical Immunology, Aarhus University Hospital, Denmark; e Research Unit of Rheumatology, Department of Clinical Research, University of Southern Denmark, Odense University Hospital, Odense, Denmark; f Center for General Practice, Department of Public Health, University of Copenhagen, Denmark; g Department of Veterinary Clinical Sciences, University of Copenhagen, Denmark; h Department of Orthopedic Surgery, Copenhagen University Hospital – Rigshospitalet, Denmark

**Keywords:** Knee, NGAL, Osteoarthritis, Biomarkers, Obesity, Weight loss, Liraglutide

## Abstract

**Objective:**

Obesity is a major risk factor for osteoarthritis (OA). Adipose tissues may be linked to OA development through secretion of potential proinflammatory cytokines including neutrophil gelatinase-associated lipocalin (NGAL). Our objective was to assess changes in serum NGAL after a low-calorie diet (LCD) and subsequent glucagon-like peptide 1 receptor agonist (GLP-1 RA) treatment.

**Design:**

A secondary analysis of a randomized, double-blinded, placebo-controlled trial in adults with overweight (BMI≥27 ​kg/m^2^) and symptomatic, early-to-moderate radiographic knee OA. Prior to randomization, participants underwent an 8-week LCD (week −8 to 0). Participants who lost min. 5 ​% of initial bodyweight were randomized 1:1 to liraglutide 3 ​mg/d or placebo for 52 weeks. Main outcome was change in serum NGAL from enrollment (week −8) to randomization (week 0). Other outcome was change in serum NGAL from week 0 to week 52 comparing liraglutide and placebo.

**Results:**

168 participants were enrolled to the initial intensive diet intervention; 127 participants, with NGAL samples, were randomized. Following the 8-week diet intervention, NGAL concentrations rose by 93.0 ​ng/mL (95 ​% CI: 18.9 to 167.1, *P* ​= ​0.015), with no correlation to weight loss magnitude. 52 weeks of treatment with either liraglutide or placebo, liraglutide did not cause a greater decrease in serum NGAL (14.9 ​ng/ml, 95%CI: −92.1 to 121.7 ​ng/mL, *P* ​= ​0.78).

**Conclusion:**

An intensive 8-week calorie restriction was associated with a rise in serum NGAL. Compared to placebo, 52 weeks of liraglutide did not cause additional changes in NGAL. This indicates a complex pattern of proinflammatory cytokine-release during hypocaloric diet interventions.

**Trial registration:**

Clinicaltrials.gov: NCT02905864.

## 1 Introduction

*Obesity is associated with a state of chronic systemic low-grade inflammation*. Adipose tissues are capable of secreting a wide variety of adipose-derived factors – collectively termed “adipokines” [1]. One of the major risk factors for osteoarthritis (OA) is obesity [2], and the observation of a possible increased risk for obese individuals to develop OA in non-weight bearing joints (hand OA) compared to lean subjects suggests the importance of adipokines in the development and progression of OA [3,4]. Proinflammatory adipokines contribute to the so-called “low-grade inflammatory state” found in overweight or obese individuals. A state which contributes to metabolic syndromes such as prediabetes and cardiovascular disease [5]. Neutrophil gelatinase-associated lipocalin (NGAL), also called lipocalin-2 (LCN2), provides a possible connection between low-grade inflammation caused by inflamed adipose tissues and OA [[6], [7], [8], [9], [10]]. NGAL has previously been associated with various inflammatory joint disease as well as septic inflammation in both animal and human joints [[11], [12], [13], [14]]. Murine studies show that white adipose tissues are a significant source of NGAL, and that NGAL is linked to key components of metabolic syndrome e.g., insulin resistance [6,8]. NGAL may also contribute to the pathophysiology of osteoarthritis through its involvement in inflammatory processes and cartilage turnover [8,15]. Evidence shows that NGAL enhances catabolic processes, promotes cartilage breakdown by binding to and blocking auto-degradation of cartilage degrading matrix metalloproteinase 9, and reduces chondrocyte viability [9,16,17]. Epidemiological evidence shows a decreased risk of knee OA after weight loss, and once developed, weight loss in obese individuals may relieve symptoms of knee OA [18,19]. While this may be explained by a simple reduction of the stress on the joint, a decrease in the proinflammatory cytokines may also be involved [20].

Liraglutide, a glucagon-like peptide 1 receptor agonist (GLP-1 RA), has a dual indication for treatment of both type 2 diabetes mellitus (T2DM) and weight loss treatment. A systematic review and meta-analysis from 2021 showed lowered c-reactive protein (CRP) and tumor ​necrosis ​factor alpha (TNF-α) levels in participants receiving a GLP-1RA compared to participants receiving either standard diabetes treatments or placebo, which suggests certain anti-inflammatory properties of GLP-1RAs [21]. From 2016 to 2019, we tested the response to liraglutide on pain and function of obese individuals with knee OA in a randomized controlled trial called the LOSE-IT trial [22]. Adult participants with obesity, or overweight, and knee OA were offered an initial 8-week low-calorie diet (LCD). Participants who had lost at least 5 ​% of initial body weight, were then randomized to either liraglutide 3 ​mg/day or placebo for 52 weeks. After 52 weeks of treatment, liraglutide induced a small additional weight loss, compared to the placebo group, but this was not accompanied by a significant difference in self-reported knee pain or joint function between the groups [22]. Based on the presumption that obesity-related worsening of OA symptoms and progression is associated with the low-grade inflammation as shown by increased NGAL-levels, we hypothesized that NGAL-levels would decrease in response to both the initial weight loss treatment and the subsequent GLP-1 RA treatment. Using data from the LOSE-IT trial, our primary aim was to measure the NGAL response to the initial 8-week LCD induced weight loss and then, secondarily, to the subsequent randomized 52-week liraglutide 3 ​mg vs. placebo weight-loss maintenance period.

## 2 Methods

### 2.1 Trial design

The study is an exploratory secondary outcome analysis from the LOSE-IT trial [22,23]. The original study was a single-center, randomized, placebo-controlled parallel-group study. Participants, investigators, and outcome-assessors were blinded. The study was conducted at the Osteoarthritis Outpatient Clinic at the Parker Institute, Copenhagen University Hospital – Bispebjerg and Frederiksberg from 2016 to 2019. The trial was prospectively registered at clinicaltrial.gov (NCT02905864).

Prior to randomization, enrolled participants started with an 8-week intensive diet weight loss program. The program included weekly dietetic counseling and the Cambridge Weight Plan’s low-calorie formula meal replacement diet (800–1000 ​kcal/day) [22]. To increase adherence participants had planned weekly facility-based group sessions, of 6–8 participants, with a dietician. The following eight weeks after randomization regular meals were partially reintroduced with daily energy intake aimed at 1200 ​kcal/day. In this period participants would also use formula diet products under the direction of a dietician. From week 8 to week 52 daily energy intake was aimed at 1500 ​kcal/day with the possibility of self-administered 1 to 2 daily meal replacements with a formula diet [22].

Participants that achieved a minimum 5 %, reduction of initial body weight after 8 weeks of intensive diet intervention, were eligible to be randomly assigned to receive either liraglutide or placebo for 52 weeks. Liraglutide, or an identical placebo, start dose was 0.6 ​mg/d and was followed by incremental biweekly dosage escalation steps of 0.6 ​mg/d that reached an end dose of liraglutide 3 ​mg daily [22]. Participants were seen every four weeks from week 8 to week 52. At these visits they would report patient-reported outcomes, receive study medication, and the first 8 weeks after randomization (week 8–16), they received meal replacements at these visits.

### 2.2 Participants

Participants between the ages of 18 and 74 years with overweight [body mass index ≥27 ​kg/m^2^], symptomatic knee OA, early-to moderate knee OA on radiography, and stable body weight were eligible for enrolment. Individuals with ongoing or recent participation in organized weight loss programs, current or recent use of medications that may cause weight loss or gain, and end-stage knee OA on radiography were ineligible for enrolment. In- and exclusion criteria as described in the original trial suppl. material [22]. In addition to these criteria, we required: NGAL data at enrollment (week −8) and randomization (week 0). Please see Ref. [22] for all eligibility criteria.

### 2.3 Randomization, allocation, and blinding

Participants were stratified by sex (male or female), age (above or below 60 years), and obesity class (BMI above or below 40 ​kg/m2) at the time of enrollment (week −8). Patients were randomized at week 0 and allocated to one of the two treatment arms. Randomization was done according to a computer-generated random assignment sequence [22]. All investigators, as well as clinical, academic, and administrative personnel, were blinded throughout the entire trial. For a full description of randomization, allocation and blinding please refer to original protocol or article [22,23].

### 2.4 Outcomes

*Main outcome:* Changes in serum NGAL concentration following the 8-week intensive weight loss period (week −8 to week 0). *Other outcomes:* Changes in serum NGAL concentration in both liraglutide and placebo arm from week 0 to week 52. *Exploratory outcomes:* Changes in [*i*] CRP, [*ii*] Total Cholesterol (TC), [*iii*] High-Density Lipoprotein (HDL), [i*v*] Low Density Lipoprotein (LDL), [*v*] Triglyceride (TG), [*vi*] Fasting Plasma Glucose (FPG), [*vii*] Hemoglobin A1c (HbA1c), [*viii*] Creatinine, [*ix*] estimated Glomerular Filtration Rate, [x] Alanine Aminotransferase (ALAT), [*xi*] Systolic blood pressure, [*xii*] Diastolic Blood Pressure: enrollment to baseline (week −8 to week 0) and baseline to study end (week 0 to week 52).

Body weight and vital signs were measured and assessed by trained healthcare professionals at enrollment, randomization and every two week afterwards as described in the original study protocol [23].

Blood samples were collected from fasting participants at weeks −8, 0, and 52 [23], and processed by trained laboratory professionals. CRP, TC, HDL, LDL, TG, FPG, Creatinine, and ALAT were analyzed using a Cobas® C 700. HbA1c samples were analyzed on a Tosoh® G8.

### 2.5 Serum measurement of NGAL

Whole blood was centrifuged at 2000g for 15 ​min, and serum was transferred to cryotubes, which were stored at −80 ​°C until further analysis. Serum NGAL concentration (ng/ml) was quantified using a commercially available electrochemiluminescence assay, R-Plex Human NGAL/LCN2 Assay ® produced by Meso Scale Discovery Inc, Rockville, Maryland, USA. Analyses were run according to the manufacturer’s instructions. A dilution factor of 500 was determined in an initial pilot run to provide NGAL measurements within instrument detection range. All samples were run as duplicates. NGAL concentrations were calculated using standardized concentration curves created with known concentrations. Mean CV between duplicates were 5.49 ​% (IQR: 1.96–7.47 ​%) for all NGAL samples (*n* ​= ​375). Five samples exceeded 20 ​% CV limit set by manufacturer. The average NGAL concentration for the duplicates was included for the analysis.

### 2.6 Statistical methods

A separate power calculation has not been performed for this study, as the number of participants with NGAL samples available was fixed and determined by completion of the trial period during the original trial. The original trial sample size of 150 participants was set to “provide a reasonable power (>80 ​%) to detect a 5 ​kg difference in body weight change between the groups, and an 8-units difference in the KOOS pain” [23]. All applicable statistical tests are two-sided and have been performed using a 5 ​% significance level. All confidence intervals presented are 95 ​% and two-sided. *Analyses populations:* We have analyzed all participants randomized at week 0 regardless of their eligibility or adherence during the original trial, however NGAL data had to be available at enrollment (week −8) for participants to be included, and therefore we have a modified intention to treat population based on the original ITT population in the LOSE-IT trial.

Statistical analyses were performed in accordance with the statistical analyses plan (see supplementary materials), which was closed before any data were analyzed. Categorical data are reported by number and percentages, while continuous data are reported by mean and SD, however median and interquartile range are reported for non-normally distributed data. The effect of the initial 8-week dietary intervention on the main outcome variable, serum NGAL, was analyzed using paired *t*-test. Crude mean change was estimated together with the associated 95 ​% confidence interval and *P*-value corresponding to the test of the hypothesis of no difference between time points (i.e., the null hypothesis). Studentized residual plots scattered against predicted values were applied in the assessment of the assumptions of a reasonable normal distribution, homoscedasticity and the absence of non-linear patterns in a general linear model. The effect of liraglutide and placebo (week 0–52) on the secondary endpoint change in serum NGAL was determined using an analysis of covariance model including a factor for treatment group and adjusted for stratification factors recorded at enrollment ([*i*]Sex, [ii] Age category and [iii] Obesity Class), as well as the level of the outcome at baseline (week 0). From this model, the observed difference in least squares means for change between liraglutide treatment and placebo was estimated together with the associated 95 ​% confidence interval and the *P*-value corresponding to the test of the hypothesis of no difference between treatments (i.e., the null hypothesis). Missing data, between week −8 and 0, was handled using multiple imputations assuming data missing at random. Multiple imputation was performed by creating five datasets using predictive mean measuring. Pooling was done according to Rubin’s Rule.

Ancillary *post hoc* analyses of the correlations between change in serum-NGAL concentrations and body weight from enrollment (week −8) to randomization (week 0), and from randomization (week 0) to follow up (week 52) were performed using Spearman’s rank correlation on the observed data (i.e., without imputation of missing data). Due to the explorative design of this study, we have not adjusted for multiplicity in this study. The interpretation of *P*-values has been based on the hierarchy of which these will be analyzed as indicated by the order of outcomes in the objectives.

## 3 Results

### 3.1 Population

**Fig. 1:**
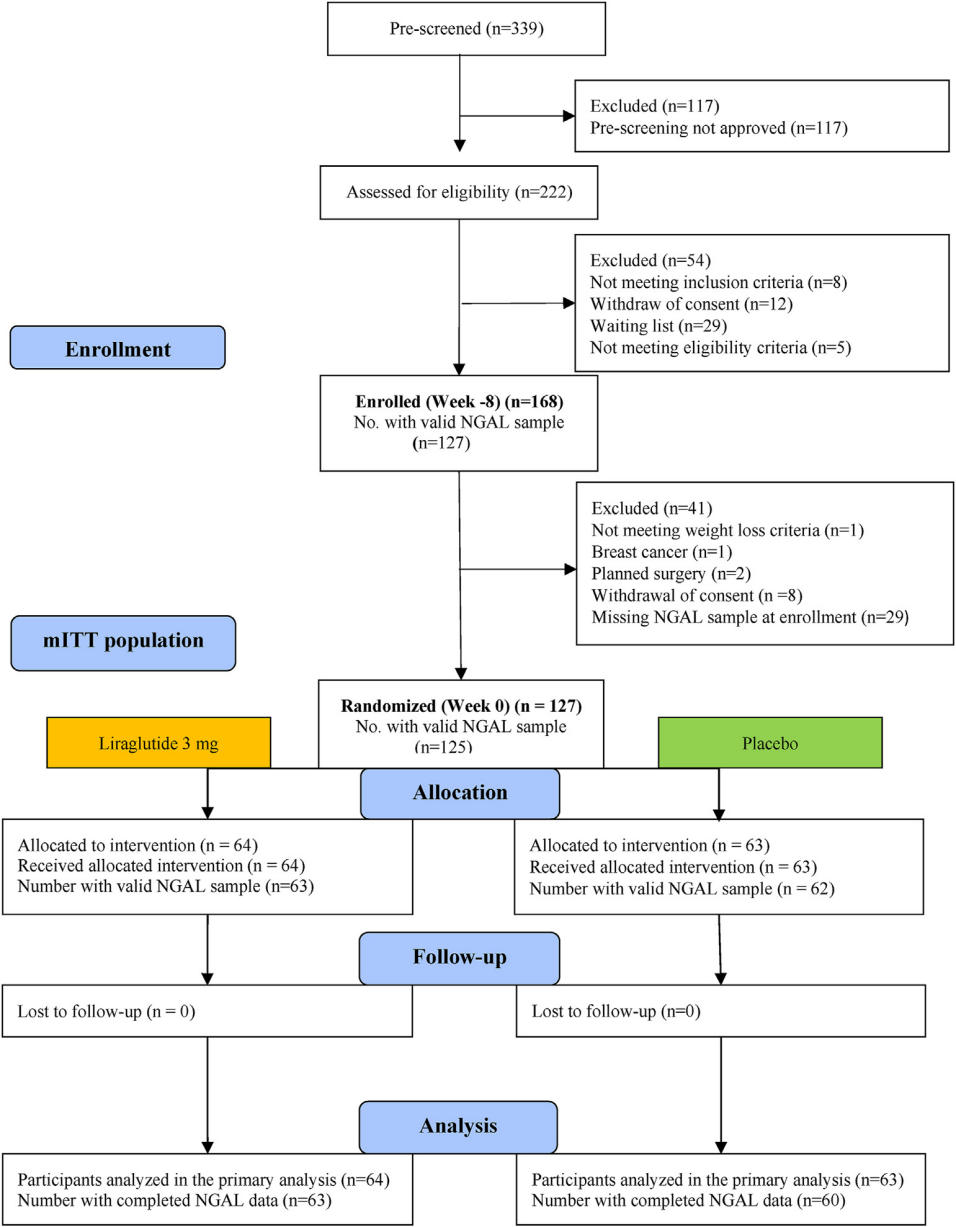
Flow diagram of study design and participants’ flow. Participants were enrolled in a low caloric dietary intervention from week −8 to week 0. Participants who achieved a successful weight loss of minimum 5 ​% of initial body weight and had a valid NGAL-sample from week −8 were included in this secondary analysis. Participants were randomized at week 0. Participants received either liraglutide (3 ​mg/d) or placebo for 52 weeks (week 0–52). Serum NGAL samples were collected at week −8, 0 and 52. NGAL, Neutrophil gelatinase-associated lipocalin.

Fig. 1 shows a flowchart illustrating the study design, participant flow, study milestones, and participation. 168 participants were enrolled in the pre-random dietary intervention from enrollment (week −8) to randomization (week 0). 12 participants were excluded due to weight loss< 5 ​%, planned surgery, malignancy, or withdrawal of consent. 29 participants did not have NGAL measurements at enrollment (week −8) and were excluded. In total 127 participants with valid NGAL measurements at enrollment were randomized (week 0), 64 and 63 participants were allocated to the liraglutide and placebo groups, respectively.

The average age at enrollment was 60 years with 84 (66 ​%) of participants being women. Among the participants, 10 (8 ​%) had known non-insulin dependent T2DM. We used participants' prescription lists to determine if participants had known cardiovascular (CVD) or chronic kidney disease and found 43 ​% of participants used medicine that could signify CVD, which seemed more prevalent in the liraglutide group (see Table 1). A detailed description of adverse, serious adverse and treatment-emergent adverse events was reported for the original trial [22].

**Table 1:** Baseline characteristics of the participants at enrollment 8 weeks prior to randomization (the mITT Population).

	All (*n* ​= ​127)	Liraglutide 3 ​mg^b^ (*n* ​= ​64)	Placebo^b^ (*n* ​= ​63)
Female sex, n (%)	84 (66)	43 (67)	41 (65)
Age, years	60 (9)	60 (9)	60 (9)
Diabetes, n (%)	10 (8)	6 (9)	4 (6)
CVD, n (%)	54 (43)	34 (53)	20 (32)
CKD, n (%)	0 (0)	0 (0)	0 (0)
Height, cm	170 (9)	171 (8)	170 (10)

Body weight, kg	106 (19)	109 (21)	102 (15)
BMI, kg/m^2^	36 (6)	37 (6)	35 (5)
Systolic blood pressure, mmHg	137 (16)	138 (17)	136 (16)
Diastolic blood pressure, mmHg	84 (10)	84 (10)	83 (10)

*Laboratory variables:*			
NGAL, ng/mL^a^	405.8 (274.8; 621.6)	432.0 (294.2; 601.7)	374.3 (256.8; 668.9)
CRP, mg/L	4.7 (4.4)	4.8 (3.8)	4.5 (5.0)
TC, mmol/L	5.5 (1.0	5.5 (1.0)	5.5 (0.9)
HDL, mmol/L	1.5 (0.4)	1.5 (0.4)	1.4 (0.4)
LDL, mmol/L	3.3 (0.9)	3.3 (0.9)	3.2 (0.9)
TG, mmol/L	1.7 (0.8)	1.6 (0.6)	1.8 (0.9)
FPG, mmol/L	6.1 (1.2)	6.2 (1.4)	6.0 (1.0)
HbA1c mmol/mol	40.0 (6.3)	40.8 (7.3)	39.2 (4.9)
Creatinine, mmol/L	76.3 (12.9)	75.2 (13.0)	77.5 (12.7)
eGFR, mL/min	81.3 (11.4)	82.4 (11.8)	80.3 (11.0)
ALAT, U/L	30.9 (17.1)	30.8 (18.4)	31.0 (16.0)

Values are mean (SD) unless otherwise indicated. CVD: Cardiovascular disease, CKI: Chronic Kidney Insufficiency – both are determined by record prescription medication, BMI: Body Mass Index, NGAL: Neutrophil gelatinase-associated lipocalin, CRP: C-Reactive Protein, TC: Total Cholesterol HDL: High-Density Lipoprotein, LDL: Low Density Lipoprotein, TG: Triglyceride, FPG: Fasting Plasma Glucose, HbA1c: Hemoglobin A1c, eGFR: estimated Glomerular Filtration Rate, ALAT: Alanine Aminotransferase.

a Median (IQR).

b Patients were not allocated into groups until week 0, hence, the groups did not formally exist from enrollment and until the end of the run-in diet period (week −8 to 0).

### 3.2 Enrollment (Week −8) to randomization (Week 0)

**Fig. 2:**
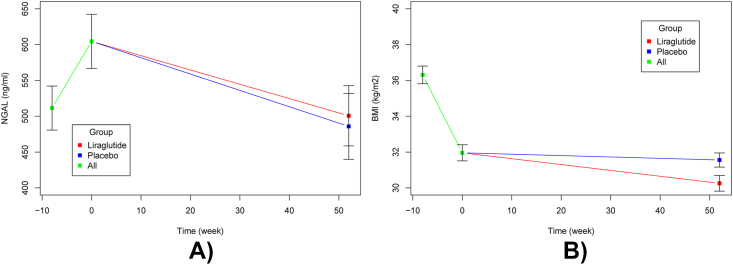
Serum NGAL **(A)** and BMI **(B)** trajectories at week −8, 0 52. Serum NGAL and BMI values at week −8 are based on crude means. Values at week 0 are estimated change between week −8 and 0, determined using a paired *t*-test for NGAL and BMI, respectively. Points at week 52 are least square means (LSM) estimating the effect of liraglutide and placebo (week 0–52) on change in serum NGAL and BMI. LSM were determined using an analysis of covariance model, that included a factor for treatment group and was adjusted for stratification factors (Sex, Age category, Obesity Class) as well as the level of the outcome at baseline (week 0). Error bars are ±1 ​× ​S.E. NGAL, Neutrophil gelatinase-associated lipocalin; BMI, Body Mass Index.

Following the initial 8-week weight loss period, participants had a mean weight loss of −12.7 ​kg (95%CI: −13.3 to −12.0) corresponding to 4.4 ​kg/m^2^ (95 ​% CI -4.5 to −4.2) as previously reported [22]. On average NGAL concentrations increased with 93.0 ​ng/mL (95%CI: 18.9 to 167.1) from a mean concentration of 511.4 ​ng/mL at enrollment corresponding to an 18 ​% increase (see Fig. 2). Nearly all our exploratory variables, including CRP, show a decrease following weight loss (Table 2).

**Table 2:** Changes from week −8 to week 0 (the mITT Population).

Variables	*N*	Change	95%CI	*p*-value
NGAL, ng/mL	125	93.0	18.9 to 167.1	0.015

CRP, mg/L	126	−1.6	−2.2 to −1.0	<0.001
TC, mmol/L	126	−1.2	−1.3 to −1.1	<0.001
HDL, mmol/L	126	−0.2	−0.2 to −0.1	<0.001
LDL, mmol/L	126	−0.8	−0.9 to −0.7	<0.001
TG, mmol/L	126	−0.6	−0.7 to −0.4	<0.001
FPG, mmol/L	126	−0.6	−0.8 to −0.5	<0.001
HbA1c, mmol/mol	125	−2.1	−2.8 to −1.5	<0.001
Creatinine, μmol/L	126	−1.8	−3.3 to −0.4	0.011
eGFR, mL/min	126	2.0	0.4 to 3.6	0.016
ALAT, U/L	125	12.2	5.6 to 18.8	<0.001

Systolic arterial pressure, mmHg	127	−9.1	−11.8 to −6.3	<0.001
Diastolic arterial pressure, mmHg	127	−5.2	−6.9 to −3.5	<0.001

Body weight, kg	127	−12.7	−13.3 to −12.0	<0.001
BMI, m/kg^2^	127	−4.4	−4.5 to −4.2	<0.001

Values are reported as means with 95 ​% CIs unless otherwise indicated. Missing data has been handled using multiple imputation (assuming ‘MAR’). NGAL: Neutrophil gelatinase-associated lipocalin, CRP: C-Reactive Protein, TC: Total Cholesterol, HDL: High-Density Lipoprotein, LDL: Low Density Lipoprotein, TG: Triglyceride, FPG: Fasting Plasma Glucose, HbA1c: Hemoglobin A1c, eGFR: estimated Glomerular Filtration Rate, ALAT: Alanine Aminotransferase, BMI: Body Mass Index.

### 3.3 Randomization (Week 0) to follow up (Week 52)

Following 52 weeks of either liraglutide or placebo treatment, the liraglutide group had a mean weight loss of −4.8 ​kg (95 ​% CI: −7.4 to −2.3), while participants of the placebo group had a weight loss of −1.1 ​kg (95 ​% CI: −3.6 to 1.5) giving a group difference of −3.8 (95%CI: −6.7 to −0.9) – similar to the main report of the trial [22]. Both groups showed an average decrease in NGAL concentrations; −103.8 ​ng/mL (SE: 41.9) for the liraglutide and −118.6 ​ng/mL (SE: 46.0) for the placebo group. The group difference in change between serum NGAL concentration at randomization and follow-up was 14.9 ​ng/ml (95%CI: −92.1 to 121.7 ​ng/mL). See Table 3 and Fig. 2A.

**Table 3:** Changes from week 0 to week 52.

Variables	Liraglutide 3 ​mg (*n* ​= ​63)	Placebo (*n* ​= ​60)	Group difference (95%CI)	*p*-value
NGAL, ng/mL	−103.8 (41.9)	−118.6 (46.0)	14.9 (−92.1 to 121.7)	0.784

CRP, mg/L	−0.7 (0.4)	−0.3 (0.5)	−0.4 (−1.5 to 0.68)	0.459
TC, mmol/L	0.7 (0.1)	0.7 (0.1)	−0.0 (−0.3 to 0.2)	0.816
HDL, mmol/L	0.3 (0.0)	0.2 (0.0)	0.1 (0.0–0.1)	0.098
LDL, mmol/L	0.4 (0.1)	0.4 (0.1)	0.0 (−0.2 to 0.2)	0.805
TG, mmol/L	0.1 (0.1)	0.3 (0.1)	−0.2 (−0.4 to 0.0)	0.061
FPG, mmol/L	0.3 (0.1)	0.4 (0.1)	−0.1 (−0.3 to 0.1)	0.274
HbA1c mmol/mol	−4.7 (0.3)	−2.5 (0.4)	−2.1 (−3.0 to −1.35)	<0.001
Creatinine, μmol/L	1.5 (1.3)	1.8 (1.4)	−0.3 (−3.6 to 2.9)	0.836
eGFR, mL/min	−0.8 (1.3)	−1.9 (1.4)	1.0 (−2.2 to 4.2)	0.537
ALAT, U/L	−15.7 (1.0)	−16.4 (1.1)	0.98 (−1.5 to 3.5)	0.443

Systolic arterial pressure, mmHg	4.4 (1.9)	4.8 (2.1)	−0.4 (−5.4 to 4.5)	0.862
Diastolic arterial pressure, mmHg	1.7 (1.2)	2.4 (1.2)	−0.7 (−3.6 to 2.3)	0.654

Body weight, kg	−4.8 (1.3)	−1.1 (1.3)	−3.8 (−6.7 to – 0.9)	0.010
BMI, kg/m^2^	−1.7 (0.4)	−0.4 (0.4)	−1.3 (−2.3 to −0.3)	0.013

Values are reported as means with 95 ​% Cis unless otherwise indicated. NGAL: Neutrophil gelatinase-associated lipocalin, CRP: C-Reactive Protein, TC: Total Cholesterol, HDL: High-Density Lipoprotein, LDL: Low Density Lipoprotein, TG: Triglyceride, FPG, Fasting Plasma Glucose, HbA1c Hemoglobin A1c, eGFR: estimated Glomerular Filtration Rate), ALAT: Alanine Aminotransferase, BMI: Body Mass Index.

For exploratory outcomes we found no significant difference between the two groups in concentration changes of lipids, plasma glucose levels (FPG), kidney function markers (creatinine and eGFR), or ALAT (Table 3). However, the liraglutide group did have a significantly larger decrease in HbA1c concentrations compared the placebo group (Table 3).

### 3.4 Post hoc analyses

#### 3.4.1 Association between weight loss and change in serum NGAL

**Fig. 3:**
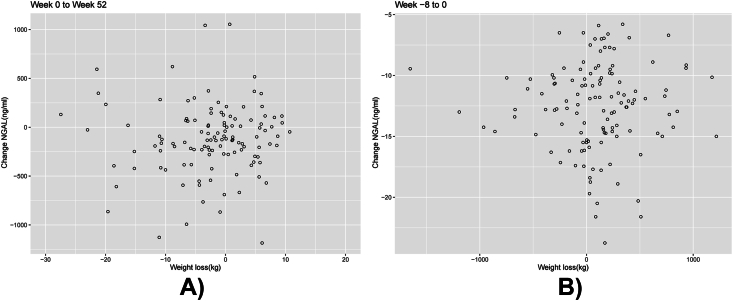
Scatterplot to illustrate association between change in serum NGAL and change in body weight from **a)** week −8 to 0 and **b)** week 0–52. NGAL, Neutrophil gelatinase-associated lipocalin.

The changes in body weight from week −8 to 0 were not correlated with the corresponding changes in serum NGAL, *ρ* ​= ​0.041 (95%CI: −0.12 to 0.20), *P* ​= ​0.65. Nor were the changes in body weight and serum NGAL, from week 0 to week 52, correlated, *ρ* ​= ​0.085 (95%CI -0.10 to 0.27), *P* ​= ​0.35; Fig. 3.

#### 3.4.2 Sensitivity analysis of duplicate CV above 20 ​%

Sensitivity analysis with removal of NGAL samples with CV, between duplicates, above 20 ​% retained NGAL trend between week −8 and week 0.

## 4 Discussion

Our primary objective was to assess changes in proinflammatory adipokines after a significant (min. 5 ​%) weight loss in obese individuals with knee OA. We chose NGAL as a presumed marker of obesity-related production of proinflammatory cytokines. Mean weight loss obtained during the LCD intervention in the first part of the study was, contrary to our expectations, associated with an 18 ​% increase in NGAL concentrations and a 34 ​% decrease in CRP. After the second phase of the study, 52 weeks of treatment with either liraglutide or placebo, serum NGAL concentrations had returned to enrollment levels, and changes in concentrations were similar for the two groups. We found a slight improvement in all metabolic biomarkers after the initial 8-week LCD. Besides the expected superiority of liraglutide on HbA1C, FPG, and body weight, the two groups had equal changes in exploratory outcomes. Furthermore, we examined whether changes in NGAL concentrations were correlated to the size of the weight loss. Therefore, we performed a post hoc correlation analysis between achieved weight loss and change in serum NGAL concentration and found no correlation. This supports that NGAL is not affected by the weight loss itself but rather, perhaps, by the intense caloric restriction that may lead to increased lipolysis in participants' adipose tissues and pro-inflammatory cytokine release from the stromal-vascular fraction of said adipose tissues [[24], [25], [26]].

Earlier studies found both positive correlations between BMI, abdominal adiposity, and serum NGAL, and higher serum NGAL concentrations in adipose populations w/o metabolic complications compared to lean individuals [[27], [28], [29], [30]]. To our knowledge only three other studies have measured serum NGAL concentrations before and after a weight loss intervention [[31], [32], [33]]. Two of the three studies used either diet or diet ​+ ​exercise interventions and found no change in serum/plasma NGAL concentrations after the interventions. The diet intervention by Nakai et al. was a Dietary Approaches to Stop Hypertension diet, which cannot be compared to the LCD used in this study, while Moreno-Navarrete et al. reduced food intake by 500 ​kcal/day, which is not comparable to an LCD limited to 800–1000 ​kcal/day. Koiou et al. did show a decrease in serum NGAL concentrations after a 6-month weight loss intervention that consisted of an energy restricted diet (metabolic rate – 600 ​kcal/day). However, half the weight loss group was also treated with weight loss medication, affecting either appetite and/or fatty acid uptake [32]. When looking into changes in NGAL after an exercise intervention, we see contradicting evidence [31,34]. NGAL was decreased after a HIIT-intervention, but Nakai et al. contradicts this by finding no difference in ΔNGAL concentrations between diet, diet ​+ ​exercise, and control groups in overweight and obese participants. So, earlier evidence shows either no change or decreases in NGAL following both diet- and training-based weight loss. The setup in these earlier studies are neither similar to each other or to our setup, neither in regard to choice of weight loss intervention or follow up period, which ranged from 0 to 24 months [[31], [32], [33], [34], [35]].

Weight loss is proposed to decrease the inflammatory state; however, the exact mechanism is unknown. A very low caloric diet has been shown to decrease inflammatory cytokines like Interleukin 6 (IL-6), TNF-α, and CRP. This decrease was first seen after six months, and it was preceded by an rise in all three inflammation cytokines at zero and three months follow-up, when compared to controls [36]. The authors of the study speculated that increased demand on adipocyte lipolysis during a very low caloric diet causes activation of adipose tissue macrophages, who then releases pro-inflammatory cytokines [37]. This temporary rise of IL-6, TNF-α and CRP is similar to the temporary rise in NGAL concentrations that we see, and it is also supported by a study showing rising IL-6 after fasting [38]. The rise in serum NGAL may reflect a temporary spike in inflammatory cytokines supporting the notion of NGAL as an inflammation marker, and perhaps more specifically as a marker of the innate immune system. Through interaction between inflammatory cytokines, NGAL has been reported to affect macrophage activation, regulation, and cytokine-release and to be associated to higher levels of lipid-associated macrophages [[38], [39], [40], [41]]. The fall in CRP concentration after both the initial LCD period does suggest a lowering of systemic inflammation, but the relationship between CRP and NGAL remains to be determined. Exercise studies describe both a non-correlation and a weak correlation between NGAL and CRP change after both exercise and/or diet interventions [31,42]. It is relevant to examine NGAL concentrations in specific tissues i.e., adipose tissue biopsies, to explore localized NGAL expression as well as systemic changes.

Despite a significantly bigger weight loss in the liraglutide group, the 14.9 ​ng/ml difference in NGAL concentration change is clinically negligible and is accompanied by a large degree of uncertainty preventing reliable interpretation. The restriction of inclusion to participants who successfully achieved a weight loss of min 5 ​% of body weight from week −8 to 0, may have diminished what effect long-term weight maintenance could have had on NGAL. In alignment with this, participants reported significant changes in knee OA symptoms from enrollment to randomization, but no further significant changes in either group at the week 52 visit [22]. Previous studies have shown that weight loss above the conventional threshold of 5–10 ​% of initial body weight is required for changes in quality of life [43,44]. Difference between groups was below this conventional threshold.

Initial NGAL-levels were approx. five times higher (mean 511 ​ng/ml) than other studies, some using Meso Scale Discovery assay kits, where NGAL concentrations ranged between 50 and 200 ​ng/ml, [[45], [46], [47]]. A review of the diagnostic accuracy of NGAL in patients with acute kidney injury does, however, show interstudy variation in mean NGAL concentrations [48]. The elevated NGAL values could represent an inflammatory activity from active rheumatologic disease and obesity. Earlier studies show varying concentrations of serum-NGAL, ranging from similar to lower concentrations in rheumatologic diseases such as axial spondyloarthritis or psoriatic arthritis [13,49,50]. A study by our group found no significant differences in NGAL concentrations between individuals with psoriasis, psoriatic arthritis, or healthy controls with serum-NGAL concentrations being similar [50]. The higher NGAL concentrations reported in this study could originate from chronic low-grade inflammation caused by the participants' OA and obesity. Most likely, however, it stems from the variance between the different kits for NGAL analyses. However, because we focus on the relative change in NGAL concentration, we accept the elevated levels.

### 4.1 Strengths and limitations

Our study is based on post hoc data from a previous, rigorously conducted randomized controlled trial under external monitoring. This ensures a meticulous quality control of treatments, data collection and participants' safety. All analyses, outcomes, and hypotheses were predefined by a statistical analysis plan (see supplementary materials), thereby reducing the risk of cherry picking and ensuring that the correct method of analysis and data treatment was agreed upon in advance. The substantial weight loss that our participants underwent increased the likelihood of detecting associated biochemical changes.

However, the post hoc nature of the study creates limitations to the trial design. Analysis of NGAL samples was only performed on samples from participants, who completed the original trial. Serum analysis was restricted to NGAL, while additional inflammation markers could have substantiated our findings. Finally, the absence of a control group limits any inferences on causation.

## 5 Conclusion

Our results show that serum NGAL concentrations rose in response to a restrictive caloric diet. The cause and effect of this rise remains elusive. It could likely be caused by the severe caloric restriction itself, through lipolysis and localized leukocyte activation, rather than by the consequent weight loss. While liraglutide, a GLP-1 RA, does elicit a bigger long term weight loss compared to placebo, this weight loss does not affect serum NGAL concentrations.

## Author contributions

The authors' responsibilities were as follows—ASP, HG, MH, HB, RC, LEK: conceptualizing, designing, writing, reviewing, and approving the manuscript; SJ, ABC, AO, ZRS, MS: writing, reviewing, and approving the manuscript; ASP, MH, HB, RC: data analysis process; HG, AO, HB, ZS, MS: acquisition of study data; and all authors: read and approved the final manuscript.

## Declaration of generative AI and AI-assisted technologies in the writing process

During the preparation of this work the author used ChatGPT, OpenAI and Copilot, Microsoft in order to correct written English and grammar. After using these tools/services, the author reviewed and edited the content as needed and takes full responsibility for the content of the publication.

## Role of the funding source

The 10.13039/100014547Parker Institute, 10.13039/501100002918Copenhagen University Hospital, Bispebjerg and Frederiksberg, is supported by a core grant from the 10.13039/100001275Oak Foundation (OCAY13-309). The LOSE-IT trial was supported by 10.13039/501100004191Novo Nordisk A/S, both financially and through the delivery of active and placebo medicine, and by the Cambridge Weight Plan through the delivery of dietary supplements [22]. The funding entities had no influence on the design of the study or in the implementation, analysis, and interpretation of data.

## Conflict of interests

ASP, ZRS, MS, RC, AO, HG, STJ, ABC, HB reports no conflict of interest to disclose in relation to this article. MH is an associate editor of Osteoarthritis and Cartilage, provides consultation on scientific advisory board for Thuasne, and has received travel grants from Contura International. LEK is currently employed at LEO Pharma.
