# Supplementary material for: Effect of weight loss and liraglutide on neutrophil gelatinase-associated lipocalin levels among individuals with overweight and knee osteoarthritis: Exploratory analyses of a randomized controlled trial
Source: Osteoarthr Cartil Open. 2025 Jan 3;7(1):100562. doi: 10.1016/j.ocarto.2024.100562 (PMC11773487; doi:10.1016/j.ocarto.2024.100562)
Supplement: Multimedia component 1 [file mmc1.pdf]

Statistical analysis plan (SAP) for:

Effect of weight loss and liraglutide on neutrophil gelatinase-associated lipocalin (NGAL) levels among patients with overweight and knee osteoarthritis: Secondary analyses of a randomized controlled trial.

Asbjørn Seenithamby Poulsen[1,2], Zara Rebecca Stisen[1,2], Sabrina Mai Nielsen[1,3], Marie Skougaard[1,4,5], Robin Christensen[1,3], Anders Overgaard[1], Henrik Gudbergsen[1,6], Stine Jacobsen[7], Andreas Peter Balslev-Clausen[8], Marius Henriksen[1,2], Henning Bliddal[1,2], Lars Erik Kristensen[1,2]

1: The Parker Institute, Copenhagen University Hospital – Bispebjerg and Frederiksberg, Denmark

2: Department of Clinical Medicine, University of Copenhagen, Denmark

3: Research Unit of Rheumatology, Department of Clinical Research, University of Southern Denmark, Odense University Hospital, Odense, Denmark

4: Center of Translational Research, Bispebjerg and Frederiksberg Hospital, Copenhagen, Denmark

5: Department of Clinical Immunology, Aarhus University Hospital, Denmark

6: Center for General Practice, Department of Public Health, University of Copenhagen, Denmark.

7: Department of Veterinary Clinical Sciences, University of Copenhagen, Denmark

8: Department of Orthopedic Surgery, Copenhagen University Hospital – Rigshospitalet, Denmark

Name and contact information for the principal investigator:

Lars Erik Kristensen
The Parker Institute, Copenhagen University Hospital, Bispebjerg and Frederiksberg,
Nordre Fasanvej 57, Road 8, entrance 19,
DK-2000 Frederiksberg
Copenhagen, Denmark
Telephone: +45 38 16 41 57
Fax: +45 38 16 41 59
E-mail: lars.erik.kristensen@regionh.dk
ORCiD ID: 0000-0002-0686-7102

1 ADMINISTRATIVE INFORMATION

1.1 Title:

Effect of weight loss and liraglutide on neutrophil gelatinase-associated lipocalin (NGAL) levels among overweight patient with knee osteoarthritis (KOA): Secondary analyses for a randomized controlled trial

Trial registration:

EudraCT Number: 2015-005163-16)

ClinicalTrials.gov identifier: NCT02905864

SAP version:

1.0 (July 5th, 2023)

Protocol version:

This document has been written based on information contained in the protocol V.2, 07th July 2021. The study is a secondary analysis of the LOSE-IT trial, with the protocol (V.6; 30 January 2017) "*Effect of liraglutide on body weight and pain in patients with overweight and knee osteoarthritis: protocol for a randomized, double-blind, placebo-controlled, parallel-group, single-centre trial*" (see: BMJ Open. 2019 May 5;9(5):e024065. doi: 10.1136/bmjopen-2018-024065) [1] and results published in the journal article "*Liraglutide after diet-induced weight loss for pain and weight control in knee osteoarthritis: a randomized controlled trial" (The American Journal of Clinical Nutrition*, Volume 113, Issue 2, February 2021, Pages 314–323, https://doi.org/10.1093/ajcn/nqaa328) [2].

Roles and responsibility:

Asbjørn Seenithamby Poulsen – Lead author

Zara Rebecca Stisen – Investigator

Sabrina Mai Nielsen – Statistical analyst

Marie Skougaard – Investigator

Robin Christensen – Senior statistical advisor

Henrik Gudbergsen – Investigator

Andreas Peter Balslev-Clausen – Scientific Advisor

Marius Henriksen – Scientific Advisor

Stine Jacobsen – Investigator

Henning Bliddal – Investigator

Lars Erik Kristensen – Principal investigator

Roles and Responsibility – signatures

Principal investigator: Lars Erik Kristensen ______ 8/8-2023

Signature and date

Lead author/investigator: Asbjørn Seenithamby Poulsen ______ 6/7-2023

Signature and date

Statistical analyst: Sabrina Mai Nielsen ______ 7th of July, 2023

Signature and date

Senior statistical advisor: Robin Christensen ______ July 6, 2023

Signature and date

2 TABLE OF CONTENTS

1 ADMINISTRATIVE INFORMATION 2

1.1 Title: 2

Trial registration: 2

SAP version: 2

Protocol version: 2

Roles and responsibility: 3

Roles and Responsibility – signatures 3

2 TABLE OF CONTENTS 4

3 PURPOSE 5

4 INTRODUCTIONS 6

4.1 Background and rationale 6

4.3 Study Objectives 7

4.4 Hypotheses 7

5 STUDY METHODS 8

5.1 Trial design 8

5.2 Randomization 8

5.3 Blinding 8

5.4 Power and sample size 9

5.5 Framework 9

5.6 Statistical interim analyses and stopping guidance 9

5.7 Timing of final analysis 9

5.8 Timing of outcome assessments 9

6 STATISTICAL PRINCIPLES 9

6.1 Confidence intervals and P values 9

6.2 Analyses populations 10

7 TRIAL POPULATION 10

7.1 Screening data: 10

7.2 Eligibility 10

7.3 Withdrawal/follow-up 11

7.4 Patient characteristics 12

8 ANALYSES 12

8.1 Outcome definitions 12

8.2 Analysis methods 13

8.3 Harms and adverse events 13

8.4 Statistical software 13

9 REFERENCE LIST 14

12 TABLE AND FIGURES 15

3 PURPOSE

This statistical analysis plan (SAP) describes detailed aspects of data preparation and analysis and was set up before initiating the analyses.

4 INTRODUCTIONS

4.1 Background and rationale

The current global obesity pandemic is both the leading cause of soaring rates of metabolic diseases and important risk factor in the development of knee osteoarthritis (KOA) [3]. Moreover, obesity and overweight has been associated with a state of chronic low-grade inflammation. Efforts dedicated to the advancement of interventions for obesity and overweight have prompted an interest in adipocyte biology. Adipose tissues are now recognized as an endocrine organ capable of secreting a wide variety of adipose-derived factors – collectively termed "adipokines" [4]. Adipokines include a variety of proinflammatory factors, most of which are increased in obese individuals. Proinflammatory adipokines appear to contribute to the so-called "low-grade inflammatory state" found in overweight or obese individuals. The indication of increased prevalence of OA in the hands, i.e. non-weight bearing joints, suggests an importance of adipokines in OA [5–7]. The presence of the adipokine neutrophil gelatinase-associated lipocalin (NGAL) or lipocalin-2(LCN2) in osteoarthritic joints provides a possible connection between low grade inflammation in obese patients and increased OA. Emerging evidence shows that NGAL may contribute to the pathophysiology of KOA through its involvement in inflammatory process and cartilage turnover with earlier studies showing NGAL to stabilize cartilage degenerating enzymes [8,9]. This suggests a possible biochemical connection between obesity/overweight and OA. NGAL has emerged as a pleiotropic molecule, involved in a variety of physiological and pathophysiological processes such as metabolic homeostasis, apoptosis, infection, immune response, or inflammation with research suggesting a possible role of NGAL in the pathophysiology of rheumatic joint diseases and osteoarthritis (OA) [9,10]. Murine in vitro studies have shown that white adipose tissues (WAT) are a significant source of NGAL and are linked to key components of metabolic syndrome e.g. insulin resistance [11]. However, contrary to previous evidence, a diet/exercise study found that there was no difference in NGAL levels following diet intervention and/or exercise in overweight and obese patients participants [12]. This underlines the need for further research to determine if NGAL has an effect in the pathophysiology of OA.

Liraglutide, a newer GLP-1 analog, with a dual indication of type 2 diabetes and of weight loss, was recently tested in a population of (KOA) patients in a randomized controlled trial (RCT), i.e., the LOSE-IT trial [1,2]. In the LOSE-IT trial, adult patients with overweight or obesity and KOA were offered an initial 8-week intensive diet intervention (IDI) followed by a weight loss maintenance period in which participants who had at least attained a 5% weight loss of initial body mass were randomized to either liraglutide 3 mg or placebo for 52 weeks. Using data from the LOSE-IT trail we

wish to examine how serum NGAL responds to the initial 8-week IDI triggered weight loss and the subsequent 52-week liraglutide 3 mg vs. placebo weight-loss maintenance period.

4.3 Study Objectives

- *Primary objective:* To evaluate changes in serum NGAL in overweight individuals with KOA undergoing a significant (≥5%) weight loss from an IDI from week -8 (enrollment) to week 0 (randomization).
- *Secondary objective:* To compare the effect of liraglutide, relative to placebo on changes in serum NGAL from week 0 (randomization) to week 52 (end of trial), in individuals with KOA who have achieved a significant (≥5%) weight loss through the initial 8-week dietary intervention.
- *Exploratory objectives:*
    - To evaluate changes in [*i*] C-reactive protein (CRP), [*ii*] total-Cholesterol, [*iii*] high-density lipoprotein cholesterol (HDL), [i*v*] low-density lipoprotein cholesterol (LDL), [*v*] triglycerides (TG), [*vi*] glucose, [*vii*] glycated hemoglobin (HbA1c), [*viii*] creatinine, [*ix*] estimated glomerular filtration rate (eGFR), [x] alanine aminotransferase (ALAT), [*xi*] systolic blood pressure (BP), [*xii*] diastolic BP, [*xiii*] body weight and [*xiv*] BMI in overweight individuals with KOA who undergo a significant (≥5%) weight loss from an 8-week intensive dietary intervention.
    - To compare the effect of liraglutide, relative to placebo, on changes in [*i*] CRP, [*ii*] total-Cholesterol, [*iii*] HDL, [i*v*] LDL, [*v*] TG, [*vi*] glucose, [*vii*] HbA1c, [*viii*] creatinine, [*ix*] eGFR, [x] ALAT, [*xi*] systolic BP, [*xii*] diastolic BP, [*xiii*] body weight and [*xiv*] BMI, from baseline (week 0) to week 52, in overweight individuals with KOA who have achieved a significant (≥5%) weight loss through the initial 8-week dietary intervention.

4.4 Hypotheses

a) The weight loss observed during the 8-week IDI (from week -8 [enrollment] to week 0 [randomization]) will result in a reduction of serum NGAL in overweight subjects with KOA.
b) Liraglutide 3 mg is superior to placebo with respect to reducing serum NGAL from week 0 (randomization) to week 52 (end of trial).

5 STUDY METHODS

5.1 Trial design

The original trial design was a single-center, double-blinded, randomized, parallel-group, placebo-controlled trial. Treatment allocation was a 1:1 ratio. The trial contained two periods: All participants were initially enrolled in an 8-week IDI period (week -8 to 0) prior to randomization (see Figure 1 in Section 12: Tables and figures). If successfully achieving a minimum weight loss of 5% (baseline body weight) during IDI, participants were randomly allocated to either liraglutide 3 mg/day or an identically appearing placebo throughout the 52-week trial period (week 0 to 52). Both groups received a tapering dietary intervention (TDI) during the initial 8 weeks after randomization (week 0 to 8) [2].

5.2 Randomization

As described in original trial protocol:

Patients were randomized in a 1:1 manner to receive either 3 mg/day liraglutide or identically appearing placebo at week 0, i.e., after the initial 8-week intensive diet period. Stratified randomization was done based on sex (male vs. female), age (<60 years vs. ≥60 years), and obesity class (BMI; < 40 kg/m^2 vs. ≥ 40 kg/m^2) status at trial enrollment (week -8). A computer-generated randomization sequence was produced using SAS PROC PLAN to generate eight separate randomization schedules before any participant was enrolled, allocating participants in permuted blocks of 2 to 6 to the daily liraglutide 3 mg/day or placebo group. Allocation concealment was secure by utilizing the computer-generated allocation process (above) where the patient identifier was coupled to one of the experimental groups only if/when the physician clicked on the 'randomization button', appearing at the baseline visit (week 0, visit T0) in the eCRF system. Upon allocation to one of the two experimental groups, the patient identifier was automatically coupled to specific pens, each of them labeled by a single and unique Dispensing Unit Number (DUN) [1].

5.3 Blinding

Following randomization, the entire process was blinded for participants and investigators, clinical, academic, and administrative trial personnel.

5.4 Power and sample size

The original trial was powered as follows: The co-primary outcomes were changes in body weight and KOOS-Pain from randomization to the end of the trial, 52 weeks after randomization. With two (co-primary) efficacy endpoints in a clinical trial, the trial's primary objective is met only if a statistically significant ($p<.05$) beneficial effect is demonstrated on both endpoints. The sample size of 150 participants was designed to provide a reasonable power (>80%) to detect a 5.5 kg difference in body weight change between the groups, and an 8-units difference in the KOOS-Pain in the original trial [2].All power and sample size analyses for the original trial were conducted using 'SAS Power and Sample Size', version 3.1 (SAS Institute Inc., Cary, North Carolina) [2].

5.5 Framework

This is a superiority trial both concerning primary and secondary outcome. The null hypothesis of the primary outcome is no change in serum NGAL following weight loss. For the secondary outcome null hypothesis is that liraglutide is not different from placebo with respect to changes in serum NGAL concentration.

5.6 Statistical interim analyses and stopping guidance

Since there were no interim analyses performed in the original trial, this was not applied in this current study.

5.7 Timing of final analysis

Not applicable. Secondary analyses to an already published trial.

5.8 Timing of outcome assessments

The outcomes investigated in this trial are assessed at weeks -8, 0, and 52.

6 STATISTICAL PRINCIPLES

6.1 Confidence intervals and P values

Level of statistical significance and use of confidence intervals: All applicable statistical tests will be two-sided and will be performed using a 5% significance level. All confidence intervals presented will be 95% and two-sided.

Rationale for any adjustment for multiplicity:

Due to the explorative design of this study, we will not adjust for multiplicity in this study. P values will be reported for all analyses, while the interpretation will be based on the hierarchy of which these will be analyzed as indicated by the order of outcomes in the objectives.

6.2 Analyses populations

We will analyze all patients randomized at week 0 regardless of their eligibility or adherence during the original trial but with NGAL data available at enrollment (week -8). This is a modified intention-to-treat (mITT) population of the original ITT population in the LOSE-IT trial.

7 TRIAL POPULATION

7.1 Screening data:

Not used in the current study.

7.2 Eligibility

Inclusion criteria in the original trial as described in suppl. material *Am J Clin Nutr* 2021;**113**:314–23. doi:10.1093/ajcn/nqaa328 [2]:

- Informed consent obtained
- Clinical diagnosis of knee OA (American College of Rheumatology [ACR] criteria) with early to moderate radiographic changes (Kellgren-Lawrence [KL] grades 1, 2, or 3)
- Age ≥ 18 years and < 75 years
- Body mass index (BMI) ≥ 27 kg/m^2
- Stable body weight during the previous 3 months (< 5 kg self-reported weight change)
- Motivated for weight loss
- Achieved at least 5% weight loss during the initial 8-week IDI (assessed at allocation visit; T0)

In addition to this we required

- NGAL data at enrollment (week -8)
- Randomization (week 0)

Exclusion criteria in the original trial:

- On-going participation, or participation within the last 3 months, in an organized weight loss program (or within the last 3 months)
- Current or history of treatment with medications that may cause significant weight gain for at least 3 months before this trial
- Current use or use within 3 months before this trial of GLP-1 receptor agonist, pramlintide, sibutramine, orlistat, zonisamide, topiramate, or phentermine
- Type 1 diabetes
- Type 2 diabetes treated with glucose-lowering drugs other than metformin
- Arthroplasty in target knee joint (most symptomatic knee at screening)
- End stage disease in target knee joint (Kellgren-Lawrence grade 4)
- Immuno-inflammatory disease
- Chronic wide-spread pain
- Pregnancy or insufficient anti-conception therapy for female fertile patients
- Breast-feeding
- Estimated glomerular filtration rate (eGFR) < 60 ml/min/1.73 m^2
- Alanine aminotransferase (ALT) or aspartate aminotransferase (AST) > 3 x above upper normal range (UNR)
- Elective surgery scheduled during the trial duration period, except for minor surgical procedures
- Surgical procedures such as arthroscopy or injections into a knee within 3 months prior to enrollment
- Previous surgical treatment for obesity (excluding liposuction >1 year before trial entry)
- Thyroid stimulating hormone (TSH) outside of the range of 0.4-6.0 mIU/L
- Obesity secondary to endocrinologic or eating disorders, or to treatment with medicinal products that may cause weight gain
- Family or personal history of medullary thyroid carcinoma or multiple endocrine neoplasia type 2
- Inflammatory bowel disease
- Congestive heart failure, New York Heart Association (NYHA) class III-IV
- Diabetic gastroparesis
- History of or current diagnosis of pancreatitis (acute and/or chronic) or pancreatic cancer
- History of cancer with the exception of in-situ malignancies of the skin or cervix uteri
- History of major depressive disorder, a PHQ-9 (Patient Health Questionnaire-9) score of more than 15, or a history of other severe psychiatric disorders or diagnosis of an eating disorder
- Subjects with a lifetime history of a suicide attempt or history of any suicidal behavior within the past month before entry into the trial
- Inability to speak Danish fluently
- A mental state impeding compliance with the program
- Use of opioids or similar strong analgesics
- Allergic reactions to the active ingredients of Saxenda, such as hypotension, palpitations, dyspnea and edema

7.3 Withdrawal/follow-up

12 participants were excluded during the 8-week weight loss period between enrollment and randomization due to insufficient weight loss, malignancy, surgery, and withdrawal of consent. 14 lost to follow-up in the liraglutide arm between randomization due to non-compliance, treatment emergent adverse effects (TEAEs) or other reasons. 13 lost to follow-up in the placebo arm between randomization due to non-compliance, TEAEs, or other reasons.

7.4 Patient characteristics

Patients will be described with according to demographics, comorbidities, laboratory values and point of care (POC) measurements, separately and combined for the two randomized groups. Data will be from enrollment visit (week -8).

8 ANALYSES

8.1 Outcome definitions

Primary outcome: Changes in serum NGAL following the 8-week intensive weight loss period (week -8 to week 0). Blood samples acquired from all participants at week -8 and 0 will be analyzed to determine serum NGAL (mg/ml). Plasma samples have been assessed using a commercially available electrochemiluminescence assay: NGAL/LCN2 R-plex Assay® produced by Meso Scale Discovery Inc (MSD), Rockville, Maryland, USA.

Secondary outcomes: Changes in serum NGAL in both liraglutide and placebo arm at week 0 and 52.

Exploratory outcomes: Changes in [*i*] CRP, [*ii*] total-Cholesterol, [*iii*] HDL, [i*v*] LDL, [*v*] TG, [*vi*] glucose, [*vii*] HbA1c, [*viii*] creatinine, [*ix*] eGFR, [x] ALAT, [*xi*] systolic BP, [*xii*] diastolic BP, [*xiii*] body weight and [*xiv*] BMI, in the two periods: enrollment to baseline (week -8 to week 0) and baseline to study end (week 0 to week 52).

8.2 Analysis methods

The effect of the initial 8-week dietary intervention on the primary outcome variable serum NGAL will be analyzed using paired t-test. In the mITT population crude mean change will be estimated together with the associated 95% confidence interval and *p*-value corresponding to the test of the hypothesis of no difference between time points (i.e., the null hypothesis).

The effect of liraglutide and placebo (week 0 to 52) on the secondary endpoint change in serum NGAL will be determined using an ANCOVA model adjusted for stratification factors recorded at enrollment ([*i*]Sex, [ii] Age category and [iii] Obesity Class), and the level of the outcome at baseline (week 0). From this model, the observed differences in least squares means for change between liraglutide treatment and placebo will be estimated together with the associated 95% confidence interval and the *p*-value corresponding to the test of the hypothesis of no difference between treatments (i.e., the null hypothesis).

In the case of missing data at week 52, missing data will be handled using multiple imputations assuming data missing at random (MAR). A simple non-responder imputation (Enrollment Observation Carried Forward [EOCF]) will be done as a sensitivity analysis, assuming data missing not at random (MNAR).

8.3 Harms and adverse events

Not applicable

8.4 Statistical software

The analysis will be carried out using R version 4.1.3 (or newer).

9 REFERENCE LIST

1 Gudbergsen H, Henriksen M, Wæhrens EE, *et al.* Effect of liraglutide on body weight and pain in patients with overweight and knee osteoarthritis: Protocol for a randomised, double-blind, placebo-controlled, parallel-group, single-centre trial. *BMJ Open* 2019;**9**:1–12. doi:10.1136/bmjopen-2018-024065

2 Gudbergsen H, Overgaard A, Henriksen M, *et al.* Liraglutide after diet-induced weight loss for pain and weight control in knee osteoarthritis: a randomized controlled trial. *Am J Clin Nutr* 2021;**113**:314–23. doi:10.1093/ajcn/nqaa328

3 Bliddal H, Leeds AR, Christensen R. Osteoarthritis, obesity and weight loss: Evidence, hypotheses and horizons - a scoping review. *Obes Rev* 2014;**15**:578–86. doi:10.1111/obr.12173

4 Ouchi N, Parker JL, Lugus JJ, *et al.* Adipokines in inflammation and metabolic disease. *Nat Rev Immunol* 2011;**11**:85–97. doi:10.1038/nri2921

5 Yusuf E, Nelissen RG, Ioan-Facsinay A, *et al.* Association between weight or body mass index and hand osteoarthritis: a systematic review. *Ann Rheum Dis* 2010;**69**:761–5. doi:10.1136/ARD.2008.106930

6 Yusuf E, Ioan-Facsinay A, Bijsterbosch J, *et al.* Association between leptin, adiponectin and resistin and long-term progression of hand osteoarthritis. *Ann Rheum Dis* 2011;**70**:1282–4. doi:10.1136/ARD.2010.146282

7 Yusuf E. Metabolic factors in osteoarthritis: Obese people do not walk on their hands. *Arthritis Res Ther* 2012;**14**. doi:10.1186/ar3894

8 Gupta K, Shukla M, Cowland JB, *et al.* Neutrophil gelatinase-associated lipocalin is expressed in osteoarthritis and forms a complex with matrix metalloproteinase 9. *Arthritis Rheum* 2007;**56**:3326–35. doi:10.1002/ART.22879

9 Villalvilla A, García-Martín A, Largo R, *et al.* The adipokine lipocalin-2 in the context of the osteoarthritic osteochondral junction. *Sci Reports 2016 61* 2016;**6**:1–13. doi:10.1038/srep29243

10 Jaberi S Al, Cohen A, D'Souza C, *et al.* Lipocalin-2: Structure, function, distribution and role in metabolic disorders. *Biomed Pharmacother* 2021;**142**:112002. doi:10.1016/j.biopha.2021.112002

11 Yan Q, Yang Q, Mody N, *et al.* The Adipokine Lipocalin 2 Is Regulated by Obesity and Promotes Insulin Resistance. *October* 2007;**56**:2533–40. doi:10.2337/db07-0007.E.D.R.

12 Nakai ME, Denham J, Prestes PR, *et al.* Plasma lipocalin-2/NGAL is stable over 12 weeks and is not modulated by exercise or dieting. *Sci Rep* 2021;**11**:1–12. doi:10.1038/s41598-021-83472-x

12 TABLE AND FIGURES

Figure 1: Flow diagram of the population

Table 1: Characteristics of the participants at enrollment

Table 2: Changes from week -8 to week 0

Figure 2. Serum NGAL (panel A) BMI (panel B) at different time points

Table 3: Changes from week 0 to week 52

Appendix 1: Changes from week -8 to week 0 (the mITT Population), with missing data replaced using non-responder imputation (EOCF)

Appendix 2: Changes from week 0 to week 52, with missing data replaced using non-responder imputation (EOCF)

Figure 1. **Flow diagram of the population**

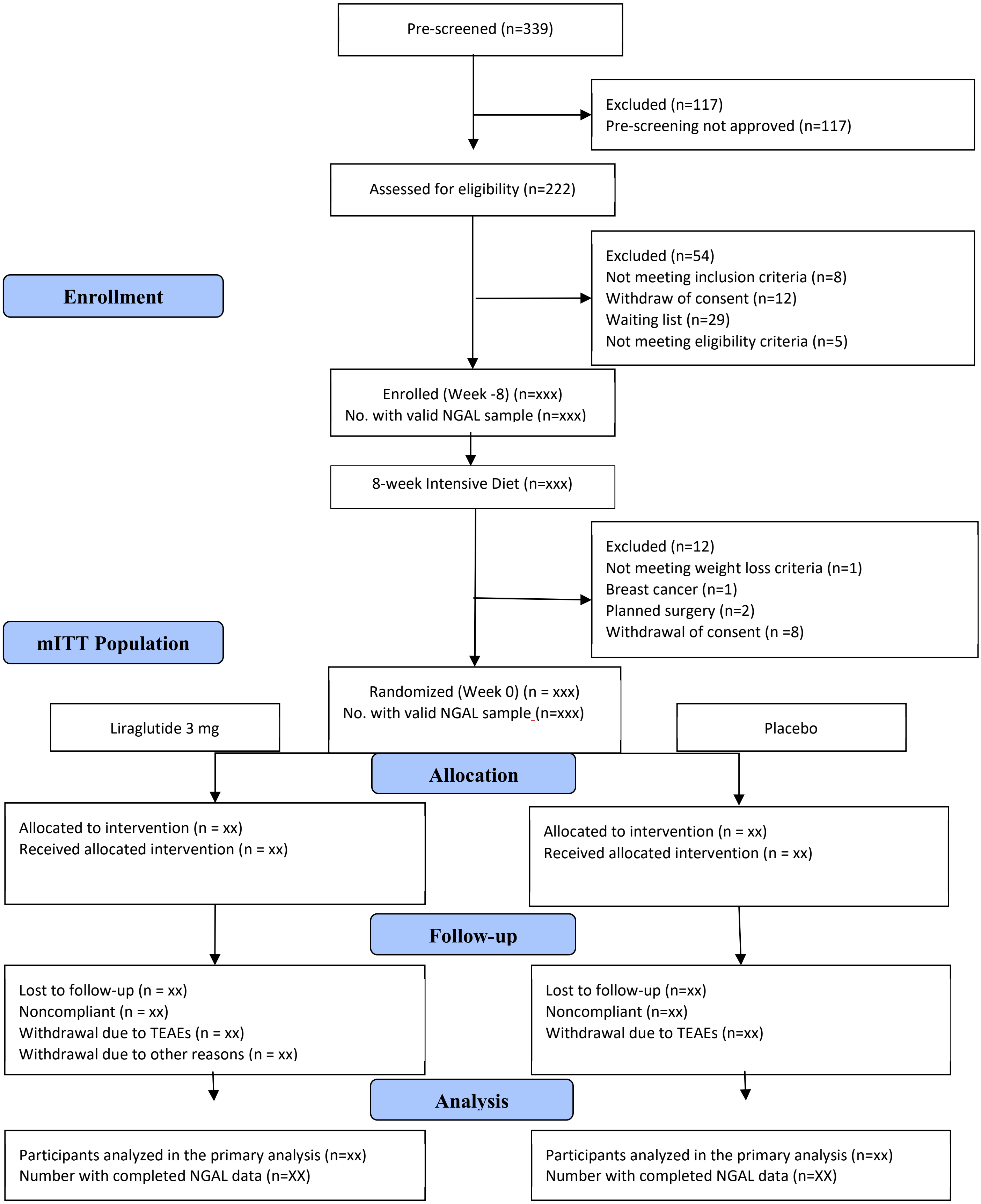

Table 1. Characteristics of the participants at enrollment 8 weeks prior to randomization (the mITT Population)

	All (n=)	Liraglutide 3 mg* (n=)	Placebo* (n=)
Female sex, n (%)			
Age, years			
Diabetes, n(%)			
CVD, n(%)			
CKD, n(%)			
Height, cm			
Body weight, kg			
BMI, m/kg^2			
Systolic Blood Pressure, mmHg			
Diastolic Blood Pressure, mmHg			
Laboratory variables:			
NGAL, ng/mL			
CRP, mg/dL			
Cholesterol, mg/dL			
HDL, mg/dL			
LDL, mg/dL			
TG, mg/dL			
Glucose mg/dL			
HBA1c mmol/ml			
Creatinine, mg/dL			
eGFR, mL/min			
ALAT, U/L			

Values are mean (SD) unless otherwise indicated. CVD: Cardiovascular disease, CKI: Chronic Kidney Insufficiency

*Patients were not allocated into groups until week 0, hence, the groups did not formally exist from enrollment and until the end of the run-in diet period (week –8 to 0)

Table 2. Changes from week -8 to week 0 (the mITT Population)

Variables	*N*	Change	95%CI	*p*-value
NGAL, ng/mL				
CRP, mg/dL				
Cholesterol, mg/dL				
HDL, mg/dL				
LDL, mg/dL				
TG, mg/dL				
Glucose mg/dL				
HbA1c mmol/ml				
Creatinine, mg/dL				
eGFR, mL/min				
ALAT, U/L				
Systolic Arterial Pressure, mmHg				
Diastolic Arterial Pressure, mmHg				
Body weight, kg				
BMI, m/kg^2				

Values will be reported as means with 95% CIs unless otherwise indicated. Missing data will be handled using multiple imputation (assuming 'MAR').

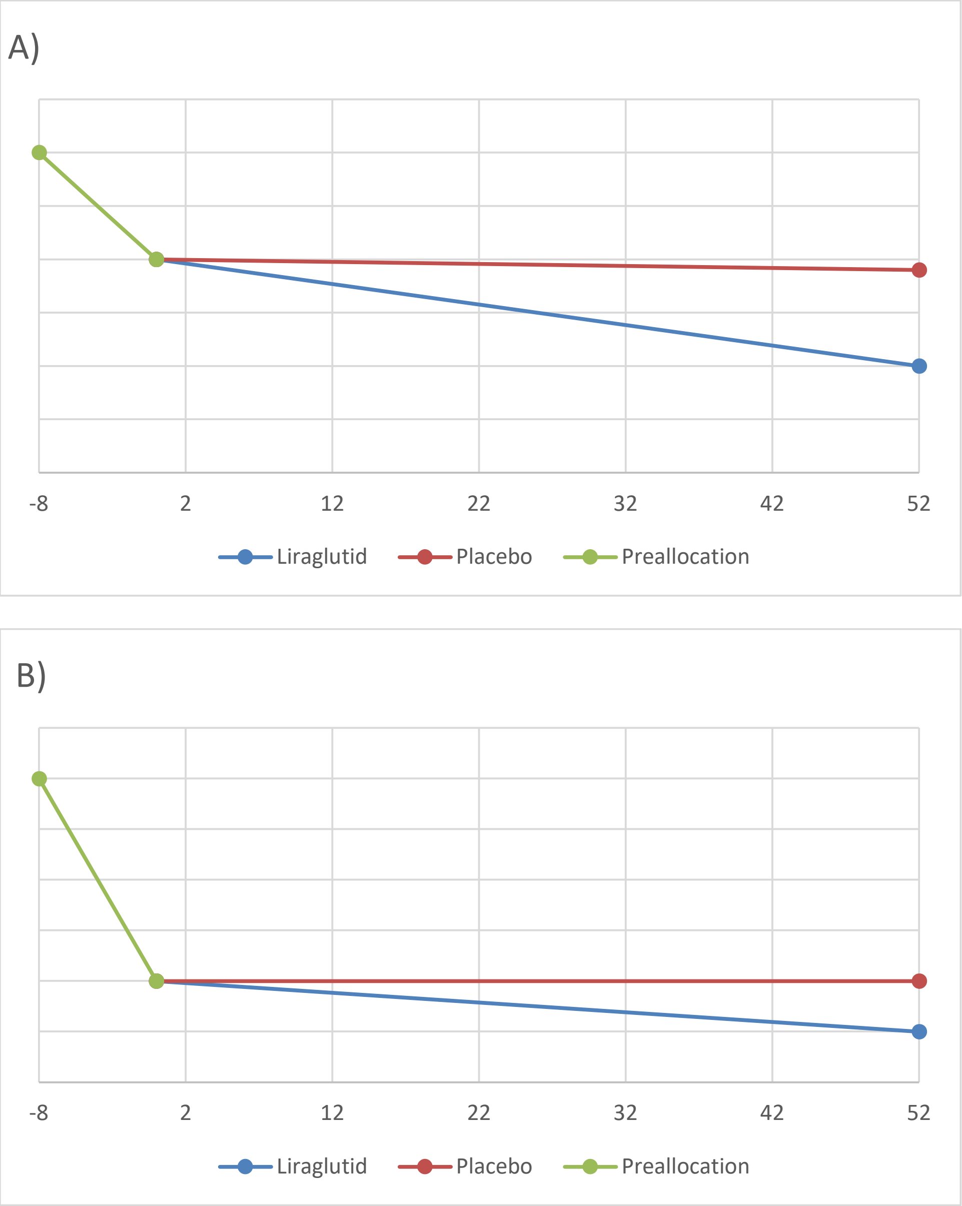

Figure 2. Serum NGAL **(panel A)** BMI **(panel B)** at different time points

(Values will be estimated based on the same models as used for table 2 and 3)

Table 3. Changes from week 0 to week 52

Variables	Liraglutide 3 mg (n=)	Placebo (n=)	Difference between groups (95%CI)	*p*-value
NGAL, ng/mL				
CRP, mg/dL				
Cholesterol, mg/dL				
HDL, mg/dL				
LDL, mg/dL				
TG, mg/dL				
Glucose mg/dL				
HbA1c mmol/ml				
Creatinine, mg/dL				
eGFR, mL/min				
ALAT, U/L				
Systolic Blood Pressure, mmHg				
Diastolic Blood Pressure, mmHg				
Body weight, kg				
BMI, kg/m^2				

Values for the groups are presented as least squares means with corresponding standard errors, and differences between groups are presented as the differences between least squares means with 95% CIs unless otherwise indicated. Missing data will be handled using multiple imputation (assuming 'MAR').

Appendix table 1. Changes from week -8 to week 0 (the mITT Population), with missing data replaced using non-responder imputation (EOCF)

Variables	*N*	Change	95%CI	*p*-value
NGAL, ng/mL				
CRP, mg/dL				
Cholesterol, mg/dL				
HDL, mg/dL				
LDL, mg/dL				
TG, mg/dL				
Glucose mg/dL				
HbA1c mmol/ml				
Creatinine, mg/dL				
eGFR, mL/min				
ALAT, U/L				
Systolic Arterial Pressure, mmHg				
Diastolic Arterial Pressure, mmHg				
Body weight, kg				
BMI, m/kg^2				

Values will be reported as means with 95% CIs unless otherwise indicated.

Appendix table 2. Changes from week 0 to week 52, with missing data replaced using non-responder imputation (EOCF)

Variables	Liraglutide 3 mg (n=)	Placebo (n=)	Difference between groups (95%CI)	*p*-value
NGAL, ng/mL				
CRP, mg/dL				
Cholesterol, mg/dL				
HDL, mg/dL				
LDL, mg/dL				
TG, mg/dL				
Glucose mg/dL				
HbA1c mmol/ml				
Creatinine, mg/dL				
eGFR, mL/min				
ALAT, U/L				
Systolic Blood Pressure, mmHg				
Diastolic Blood Pressure, mmHg				
Body weight, kg				
BMI, kg/m^2				

Values for the groups are presented as least squares means with corresponding standard errors, and differences between groups are presented as the differences between least squares means with 95% CIs unless otherwise indicated.
