# Supplementary material for: Effect of weight loss and liraglutide on neutrophil gelatinase-associated lipocalin levels among individuals with overweight and knee osteoarthritis: Exploratory analyses of a randomized controlled trial
Source: Osteoarthr Cartil Open. 2025 Jan 3;7(1):100562. doi: 10.1016/j.ocarto.2024.100562 (PMC11773487; doi:10.1016/j.ocarto.2024.100562)
Supplement: Multimedia component 2 [file mmc2.docx]

Appendix A: Description of Multiple Imputation

Missing data was handled using multiple imputations assuming data missing at random (MAR). All missing data at week 0 were from biochemical variables. Serum neutrophil gelatinase associated lipocalin (NGAL), Alanine Aminotransferase (ALAT) and Hemoglobin A1c (HbA1c) each had 2 missing values. All other laboratory values had 1 missing value at week 0. Our rigorous inclusion- and exclusion criteria, and the amount of baseline characteristics collected, gives no reason to believe that missing data occurred due to unobserved factors. Furthermore, observed data does not deviate from imputed data (see table below). Most missing laboratory data originated from one participant, who still had body weight, blood pressure and NGAL data which indicates that the participant did show up in the outpatient clinic for the week 0 visit. Because of this we assume that values were missing at random probably due to loss of blood sample or an error during automated laboratory analysis. Multiple imputation was performed by creating five datasets using predictive mean measuring. Five separate datasets were created for NGAL using predictive mean measuring. For all other laboratory values, we created five dataset using predictive mean measuring. Pooling was done according to Rubin’s Rule, where five paired t-tests were performed on imputed data. A mean estimate, and 95% confidence interval from these five tests were calculated. Imputed estimates were checked against estimates from students paired t-test performed on observed data. For all imputed values we saw a change of about 0.1mg/ml, whilst the estimate for NGAL fell 1 ng/ml and the 95% confidence interval widened with 3ng/ml (see table page 2)

Table of complete cases, week -8 to week 0

| Outcome | Estimate | 95% conf.int. |
| --- | --- | --- |
| ΔNGAL | 94.6 | 19.5 to 170.0 |
| ΔCRP | -1.7 | –2.3 to -1.1 |
| ΔTC | -1.2 | -1.3 to -1.1 |
| ΔHDL | -0.2 | -0.2 to -0.1 |
| ΔLDL | -0.8 | -0.9 to -0.7 |
| ΔTG | -0.6 | -0.7 to -0.4 |
| ΔFPG | -0.6 | -0.8 to -0.5 |
| ΔHBA1c | -2.2 | -2.9 to -1.5 |
| ΔCrea | -2.0 | -3.4 to -0.6 |
| ΔeGFR | 2.1 | 0.5 to 3.7 |
| ΔALA | 12.3 | 5.5 to 19.0 |
